# Genetic and clinical characterization of SPG10: a case series of novel pathogenic variants and phenotypic diversity

**DOI:** 10.1097/MS9.0000000000004014

**Published:** 2025-10-07

**Authors:** Abdallah N. Mansour, Georgia Karadima, Georgios Koutsis, Shadi Salloum

**Affiliations:** a Athens International Master Program in Neuroscience, National and Kapodistrian University of Athens, Athens, Greece; b Aiginitio Hospital, Athens, Greece; c Cancer Research Center, Tishreen University Hospital, Latakia, Syria; d Harvard Medical School, Boston, Massachusetts, USA

**Keywords:** autosomal dominant inheritance, axonal transport dysfunction, case series, corticospinal tract degeneration, kinesin-related disorders

## Abstract

**Introduction and importance:**

KIF5A, a kinesin family protein, plays a crucial role in intracellular transport in nerve cells. Pathogenic variants in KIF5A, such as those causing SPG10, lead to corticospinal tract degeneration, resulting in hereditary spastic paraplegia (HSP). This case series highlights a novel KIF5A variant and its clinical significance in three Greek siblings with SPG10.

**Methods:**

Three siblings with progressive gait spasticity and neurological features underwent comprehensive diagnostic evaluations, including MRI, nerve conduction studies, and genetic testing. Whole exome sequencing followed by Sanger validation identified a novel KIF5A variant (c.604C>G; p.Ser202Gly). Management strategies involved physiotherapy, occupational therapy, and Baclofen for spasticity.

**Outcomes:**

Symptom progression was observed in two siblings, while one remained stable during follow-up. Cervical spondylosis and neuropathy were noted in all cases. The identified variant disrupts microtubule dynamics, contributing to corticospinal tract degeneration.

**Conclusions:**

This is the first documented case series of SPG10 associated with a novel KIF5A variant in Greece. It underscores the importance of genetic testing for accurate diagnosis and highlights the need for continued research into SPG10 pathophysiology and targeted treatments.

## Introduction

Hereditary spastic paraplegia (HSP) comprises a genetically heterogeneous group of neurodegenerative disorders characterized by progressive lower limb spasticity and weakness, with varying additional neurological features^[1]^.

Among the numerous subtypes, SPG10 is linked to pathogenic variants in the KIF5A gene, which encodes a critical motor protein involved in intracellular axonal transport^[2]^. The degeneration of corticospinal tracts associated with these variants underlies the hallmark spastic gait observed in affected individuals^[3]^. SPG10 is typically inherited in an autosomal dominant manner, although rare instances of autosomal recessive inheritance have also been reported^[4]^.

**HIGHLIGHTS**

- Cause: SPG10, a subtype of HSP, is linked to a pathogenic variant (c.604C>G; p.Ser202Gly) in the KIF5A gene, disrupting axonal transport and microtubule function.
- Cases: Three siblings (2 brothers, 1 sister) presented with progressive spasticity, ataxia, falls, and unique features like ophthalmoplegia and ptosis.
- Treatment: Management included physiotherapy, Baclofen for spasticity, and genetic counseling for family planning.
- Significance: First SPG10 case reported in Greece, showcasing variable presentations, including Parkinson-like symptoms in one sibling.
- Future Needs: Further research into SPG10’s pathophysiology and therapies is vital to improve outcomes for affected individuals.

What makes this case series particularly educational and significant is the identification of a novel heterozygous KIF5A variant (c.604C>G; p.Ser202Gly) in three Greek siblings with SPG10. To our knowledge, this is the first reported instance of this specific variant and its association with SPG10 in Greece. These findings contribute to the expanding genetic landscape of SPG10 and highlight the importance of comprehensive genetic testing for diagnosing hereditary spastic paraplegia^[5]^. Additionally, the siblings presented with distinct clinical features, including cerebellar ataxia and asymmetric ptosis, providing valuable insights into the phenotypic variability of SPG10.

This case series aims to enhance the understanding of SPG10 pathophysiology by integrating clinical findings with genetic analysis. It underscores the crucial role of early genetic diagnosis in facilitating personalized management and guiding genetic counseling strategies for affected families^[6]^. Furthermore, the documentation of this novel variant expands current knowledge and provides a foundation for future research efforts in the field of neurogenetics^[7]^.

## Methods

Registration: This study was conducted in accordance with the Declaration of Helsinki. Due to the retrospective nature of the case series, no formal research registry number was obtained.

Study Design: The present investigation is a retrospective, single-center case series. The cases were non-consecutive and identified through a review of clinical records.

Settings and Time-Frames: The study was conducted at a tertiary neurology clinic in Greece. Data collection, including patient recruitment and diagnostic workup, spanned from January 2020 to December 2022. Follow-up assessments were conducted at regular intervals up to June 2023.

**Figure 1.**
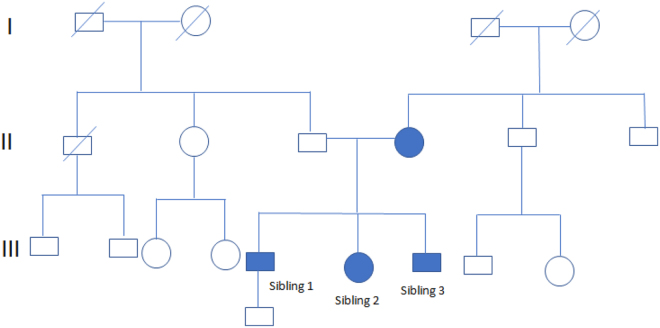
Pedigree of the family with spastic paraplegia.

Participants: The study included three Caucasian siblings (two brothers and one sister) diagnosed with hereditary spastic paraplegia type 10 (SPG10). The age of symptom onset ranged between 19 and 39 years. Clinical features included progressive spastic gait, cerebellar ataxia, and horizontal ophthalmoplegia. One sibling also presented with asymmetric ptosis. The family pedigree is depicted (Fig. 1), and participant characteristics are detailed in Table 1.

Black circles (females) and squares (males) represent affected people with spastic paraplegia. Being with a crossed line deceased, without a crossed line alive.Undiagnosed individuals are not shaded.

**Table 1:** Patient characteristics

Patient characteristics	Patient 1	Patient 2	Patient 3 index patient
Gender	Male	Female	Male
Onset (years)	39	36	19
Age(years) at presentation	44	40	31
First review date	01/03/2024	30/10/2018	01/06/2007
Symptoms at presentation	Slowly progressive difficulty in walking	Progressive difficulty in walking	Progressive difficulty in walking
		Urinary urgency with frequent episodes of incontinence	
			Urinary urgency
		Symptoms of depression and anxiety	
Past history	Bell’s palsy (2020) in the right side of his face	–	High alcohol intake during the period 2001-2004
Clinical Signs (1st visit)	Independent walking	Walks with unilateral support	Independent walking
	Brisk Jaw Jerk reflex	Brisk Jaw Jerk reflex	in the upper limbs reduced tendon reflexes
	In the upper limbs reduced tendon reflexes	Normal tendon reflexes in the upper limbs	Exaggerated lower limb reflexes and extensor plantar responses
	Exaggerated quadriceps femoris reflex and reduced triceps surae tendon reflex and extensor plantar responses	Exaggerated lower limb reflexes and extensor plantar responses	Lower limb spasticity
	Lower limb spasticity	Lower limb spasticity	Non sustained ankle clonus
	Impaired vibration and pain /pinprick sense in the lower limbs	Impaired vibration sense in the lower limbs	Impaired pinprick sense in the lower limbs
	Hammer toes, pes cavus	Mild distal muscle weakness in the upper limbs and moderate proximal and distal weakness in the lower limbs	Hammer toes, pes cavus
	No weaknesses	Bilateral 1st dorsal interosseous muscle atrophy	Distal weakness in the lower limbs
	Mild distal muscle atrophy of lower limbs	Signs of parkinsonism (hypomimia, upper limb rigidity and bradykinesia (right predominance) and mild right hand rest tremor)	
Patient concerns	Collapses and gait abnormality with tendency to fall down, Urinary urgency		
Medication		Baclofen 10 mg 1-0-1 per os	Gabapentin 300 mg 2 × 3
		Recommendation of initiation of sertraline 100 mg once a day (titrated up to 150 mg 12/2018)	Pregabalin 75 mg once a day.
			Recommendation of discontinuation of Gabapentin due to liver enzyme elevation
Second review		26/3/2019	22/7/2018
Clinical reevaluation (2nd visit)		Significant improvement of mood disorder.	Deterioration of deep sensation impairment
		Deterioration of parkinsonism.	Bilateral ptosis (eyelid)
		Recommendation of initiation of levodopa/ benserazide 1/4x3, may titrate up to 1/2x3	Ulcers in the lower limbs
Third review		11/12/2019	
Clinical reevaluation (3nd visit)		Deterioration of mood disorder after sertraline discontinuation.	
		Intensive physical therapy since 9/2019 and Botox injections (6/2019 and 10/2019) for spasticity.	
		Deterioration of parkinsonism and proximal muscle weakness of lower limbs. (still walking with unilateral support)	
Fourth review		29/12/2021	
Clinical reevaluation (4th visit)		Deterioration of walking ability. Needs bilateral support for a few steps, requires wheelchair for longer distances. Combination of pyramidal and extrapyramidal system impairment.	
Fifth review		8/11/2022	
Clinical reevaluation (5th visit)		Wheelchair bound. Suffers from trunk and jaw rigidity that she associates with the use of antiparkinsonian medication (dyskinesias?)	
		Recommendation of slow decrease of dosage of antiparkinsonian medication.	
		She is on duloxetine 60 mg 0-0-1 and pregabalin 50 mg 1-0-0	

Participants were recruited through referrals from peripheral hospitals following the unclear etiology of their symptoms. Inclusion criteria included a confirmed diagnosis of SPG10 based on genetic testing and clinical features consistent with hereditary spastic paraplegia. Patient information was de-identified to maintain confidentiality.

Pre-Intervention Patient Optimization: No specific lifestyle changes, pre-surgical preparations, or psychological support interventions were required due to the chronic nature of the disease. Medication reviews were conducted to tailor pharmacological management.

Interventions: The primary interventions consisted of non-pharmacological and pharmacological approaches, including:

Physiotherapy: To improve motor function and reduce spasticity.

Occupational Therapy: To assist with daily activities and adaptive techniques.

Pharmacological Therapy: Baclofen was prescribed to the first two siblings for spasticity management.

Intervention Details: The rationale for the prescribed interventions was to alleviate spasticity and enhance motor coordination. Baclofen was administered orally at individualized dosages determined by tolerance and response. The physiotherapy sessions focused on balance training and strengthening exercises.

Operator Details: Physiotherapy and occupational therapy were provided by licensed professionals with extensive experience in neurodegenerative disorders. Pharmacological management was overseen by the neurology clinic’s senior consultant.

Quality Control: To ensure consistency between cases, a standardized physiotherapy protocol was applied. Treatment efficacy was monitored through regular clinical assessments and patient-reported outcomes.

Follow-Up: Participants were followed up at 6-month intervals for a maximum period of 36 months. Follow-up assessments included clinical examinations and patient interviews conducted at the clinic. Post-intervention instructions included adherence to prescribed therapy regimens and regular physiotherapy sessions.

Two of the three siblings reported symptom progression during follow-up, while the third sibling remained stable. No participants were lost to follow-up. Detailed follow-up outcomes are presented in Table 2.

**Table 2:** Diagnostic information

Diagnostic test	Patient 1	Patient 2	Patient 3
MRI findings	Mild cervical spondylosis without myelopathy <br> Mild cervical disc degeneration without nerve root impingement	Mild cervical and thoracic spondylosis without myelopathy	Unremarkable MRI findings
ENG	Axonal length-dependent sensorimotor polyneuropathy	Axonal length-dependent sensorimotor polyneuropathy	Sensorimotor demyelinating polyneuropathy (probably mixed)
ENT evaluation			Evidence of moderate bilateral sensorineural deafness
IgA			448 mg/dL (high)
Free testosterone			68.4 (high)
Metabolomic profile			See metabolomic profile (organic acids analysis)
B12 malabsorption			Positive Schilling test
Skin punch biopsy			Histopathologic findings of leukocytoclastic vasculitis

The work has been reported in line with the PROCESS 2025 (www.processguideline.com) criteria^[8]^.

## Results

### Participants

The case series involved three Caucasian siblings, two males and one female, aged between 42 and 63 years at the time of the study. The age of symptom onset ranged from 19 to 39 years. Clinical features included progressive lower limb spasticity, cerebellar ataxia, and horizontal ophthalmoplegia, with the third sibling also presenting with asymmetric ptosis. One sibling reported a family history of undiagnosed ataxia. Comorbidities included cervical spondylosis and neuropathy, as identified through MRI and nerve conduction studies (Table 2).

### Deviation from the initial management plan

There were no significant deviations from the initial management plan. The prescribed interventions, including physiotherapy, occupational therapy, and Baclofen administration, were implemented as planned. Minor dosage adjustments for Baclofen were made based on individual tolerance.

### Outcomes and follow-up

During the follow-up period of 36 months, two siblings demonstrated symptom progression, including worsened gait instability and increased spasticity. The third sibling’s condition remained stable without further neurological decline. Expected outcomes based on literature suggested gradual disease progression in SPG10 cases, consistent with observed results^[9]^. No formal patient-reported measures were utilized. There was no loss to follow-up.

### Intervention adherence and compliance

All participants adhered to the prescribed physiotherapy and occupational therapy regimens. Baclofen was well-tolerated, with no reported side effects. Adherence was monitored through follow-up clinical assessments and patient self-reports.

### Complications and adverse events

No complications or adverse events were reported during the study period. Precautionary measures included regular monitoring for side effects of Baclofen and physiotherapy-related injuries. The intervention and follow-up protocols proceeded without unanticipated events, and no complications were reported to any regulatory agency.

## Discussion

### Summary of key results

**Figure 2.**
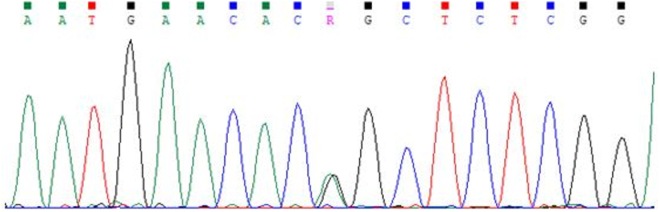
Sanger sequencing chromatogram.

This case series describes three siblings with hereditary spastic paraplegia type 10 (SPG10) linked to a novel heterozygous KIF5A variant (c.604C>G; p.Ser202Gly) (Fig. 2). Key clinical features included progressive lower limb spasticity, cerebellar ataxia, and horizontal ophthalmoplegia. One sibling also presented with asymmetric ptosis. In particular, Patient 2 exhibited a distinctive progression trajectory. Symptoms began in adolescence with subtle gait imbalance, which gradually evolved into pronounced spastic paraparesis by the third decade of life. Over subsequent years, cerebellar ataxia and horizontal ophthalmoplegia became increasingly evident, contributing to severe functional impairment. This stepwise progression underscores the slowly degenerative nature of SPG10, in line with reports that KIF5A-related HSP often follows a chronic course with cumulative neurological deficits over decades^[2,9]^. The combination of early gait instability with later cerebellar involvement and oculomotor dysfunction reflects not only corticospinal tract degeneration but also broader disruption of neuronal networks dependent on axonal transport. Recognizing such longitudinal patterns is essential for prognosis, genetic counseling, and guiding supportive therapy.

Chromatogram showing the Sanger sequencing results for the genetic analysis of *KIF5A*. The peaks represent the nucleotide sequence, with the black peak indicating the wild-type allele and the green peak representing the mutated allele. The presence of two distinct peaks confirms the heterozygous status of the mutation (c.604A>G).

### Relevant literature and context

KIF5A mutations are known to disrupt intracellular transport, contributing to corticospinal tract degeneration and neurodegeneration in hereditary spastic paraplegia^[7]^. The findings in this study align with previously reported SPG10 phenotypes but also highlight a unique presentation with asymmetric ptosis in one sibling. The role of the identified KIF5A variant in microtubule dynamics underscores its potential contribution to disease pathophysiology^[10]^. Genetic testing and counseling remain pivotal for diagnosing hereditary neurological disorders, including SPG10^[3]^. These findings support current clinical guidelines emphasizing early genetic testing for patients with hereditary spastic paraplegia. The pathophysiological mechanism underlying SPG10 involves impaired axonal transport due to mutations in the KIF5A gene, which encodes a neuronal kinesin motor protein essential for the anterograde transport of organelles and vesicles along microtubules. Disruption of this transport system leads to axonal degeneration, particularly affecting long corticospinal tracts. Recent studies suggest that altered mitochondrial dynamics and accumulation of defective organelles may also contribute to neuronal dysfunction and degeneration in SPG10^[7]^. Additionally, KIF5A mutations have been implicated in the dysregulation of synaptic transmission and neuronal survival pathways, further exacerbating neurodegeneration.

### Strengths

A notable strength of this study is its comprehensive genetic analysis, which included whole-exome sequencing, Sanger validation, and next-generation sequencing for hereditary spastic paraplegia genes. This approach ensured accurate identification of the novel KIF5A variant. Additionally, the case series contributes valuable data from a previously underrepresented population in Greece. The multidisciplinary management involving physiotherapy, occupational therapy, and pharmacological intervention highlights a holistic care approach.

### Weaknesses and limitations

The retrospective nature of the study and the small sample size limit the generalizability of the findings. Moreover, patient-reported outcome measures, such as quality-of-life assessments, were not included. The absence of functional studies to confirm the pathogenicity of the identified variant is another limitation. Future studies could explore larger cohorts to validate these findings and assess the variant’s impact on cellular processes.

### Directions for future research

This study raises important questions regarding the precise molecular mechanisms by which the KIF5A p.Ser202Gly variant affects neuronal function and contributes to SPG10 pathophysiology. Functional studies are necessary to elucidate these mechanisms. Additionally, future research could benefit from prospective, multicenter studies to explore the natural history and long-term outcomes of SPG10. Investigating therapeutic approaches aimed at enhancing axonal transport, stabilizing microtubule dynamics, or mitigating mitochondrial dysfunction may offer promising avenues for disease modification. Exploring gene-targeted therapies and neuroprotective interventions will also be crucial to improving patient care.

## Conclusion

This study highlights the diverse clinical manifestations and genetic foundations of SPG10-HSP, with a focus on the identification of pathogenic variants in the SPG10 gene through Whole Exome Sequencing (WES). The first reported case of SPG10 in Greece is included, which presents a novel KIF5A variant, contributing valuable insight into rare genetic disorders. This case reinforces the critical importance of early diagnosis and personalized management in improving patient outcomes.

The findings of this study are supported by the identification of specific genetic variants, which offer a strong rationale for the use of genetic testing as a diagnostic tool. These genetic insights are crucial for understanding the underlying mechanisms of SPG10 and its associated hereditary spastic paraplegia (HSP).

Looking ahead, further research is needed to explore the pathophysiology of SPG10 and to develop targeted therapies. Questions regarding potential therapeutic interventions and tailored management strategies for patients with SPG10 remain unanswered, highlighting the need for continued investigation in future studies. This research is essential for improving clinical practices, and sharing this case may contribute to knowledge exchange among clinicians and researchers, ultimately enhancing patient care.

## Data Availability

Not applicable.

## Research registration unique identifying number (UIN)

Not needed.

## References

[1] Fink JK. Hereditary spastic paraplegia: clinico-pathologic features and emerging molecular mechanisms. Acta Neuropathol 2013;126:307–28. 23897027 10.1007/s00401-013-1115-8 PMC4045499

[2] Schüle R Wiethoff S Martus P. Hereditary spastic paraplegia: clinicogenetic lessons from 608 patients. Ann Neurol 2016;79:646–58. 26856398 10.1002/ana.24611

[3] Lo Giudice T Lombardi F Santorelli FM. Hereditary spastic paraplegia: clinical-genetic characteristics and evolving molecular mechanisms. Exp Neurol 2014;261:518–39. 24954637 10.1016/j.expneurol.2014.06.011

[4] Blackstone C O’Kane CJ Reid E. Hereditary spastic paraplegias: membrane traffic and the motor pathway. Nat Rev Neurosci 2011;12:31–42. 21139634 10.1038/nrn2946 PMC5584382

[5] Novarino G Fenstermaker AG Zaki MS. Exome sequencing links corticospinal motor neuron disease to common neurodegenerative disorders. Science 2014;343:506–11. 24482476 10.1126/science.1247363 PMC4157572

[6] Daoud H Zhou S Noreau A. Exome sequencing reveals SPG11 mutations causing juvenile ALS. Neurobiol Aging 2012;33:839.e5–9. 10.1016/j.neurobiolaging.2011.11.012 22154821

[7] Reid E. Hereditary spastic paraplegia and amyotrophic lateral sclerosis: distinct presentations of kinesin-associated protein mutations. J Neurol Sci 2003;110:76–79.

[8] Agha RA Mathew G Rashid R. Revised preferred reporting of case series in surgery (PROCESS) guideline: an update for the age of artificial intelligence. Prem J Sci 2025;10:100080.

[9] Fereshtehnejad SM Saleh PA Oliveira LM. Movement disorders in hereditary spastic paraplegia: a systematic review and meta-analysis. Neurol Sci 2023;44:947–59. 36441344 10.1007/s10072-022-06516-8 PMC9925593

[10] Reid E Kloos M Ashley-Koch A. A kinesin heavy chain (*KIF5A*) mutation in hereditary spastic paraplegia (SPG10). Am J Hum Genet 2002;71:1189–94. 12355402 10.1086/344210 PMC385095

